# Exposure to the Methylselenol Precursor Dimethyldiselenide Induces a Reductive Endoplasmic Reticulum Stress in *Saccharomyces cerevisiae*

**DOI:** 10.3390/ijms22115467

**Published:** 2021-05-22

**Authors:** Marc Dauplais, Pierre Mahou, Pierre Plateau, Myriam Lazard

**Affiliations:** 1 Laboratoire de Biologie Structurale de la Cellule, BIOC, École Polytechnique, CNRS-UMR7654, IP Paris, 91128 Palaiseau, France; marc.dauplais@polytechnique.edu (M.D.); plateau@bioc.polytechnique.fr (P.P.); 2 Laboratoire d’Optique et Biosciences, École Polytechnique, CNRS UMR7645—INSERM U1182, IP Paris, 91128 Palaiseau, France; pierre.mahou@polytechnique.edu

**Keywords:** selenium, methylselenol, dimethyldiselenide, ER stress, reductive stress, unfolded protein response, disulfide bond formation, oxidative folding

## Abstract

Methylselenol (MeSeH) is a major cytotoxic metabolite of selenium, causing apoptosis in cancer cells through mechanisms that remain to be fully established. Previously, we demonstrated that, in *Saccharomyces cerevisiae*, MeSeH toxicity was mediated by its metabolization into selenomethionine by O-acetylhomoserine (OAH)-sulfhydrylase, an enzyme that is absent in higher eukaryotes. In this report, we used a mutant *met17* yeast strain, devoid of OAH- sulfhydrylase activity, to identify alternative targets of MeSeH. Exposure to dimethyldiselenide (DMDSe), a direct precursor of MeSeH, caused an endoplasmic reticulum (ER) stress, as evidenced by increased expression of the ER chaperone Kar2p. Mutant strains (∆*ire1* and ∆*hac1*) unable to activate the unfolded protein response were hypersensitive to MeSeH precursors but not to selenomethionine. In contrast, deletion of *YAP1* or *SKN7*, required to activate the oxidative stress response, did not affect cell growth in the presence of DMDSe. ER maturation of newly synthesized carboxypeptidase Y was impaired, indicating that MeSeH/DMDSe caused protein misfolding in the ER. Exposure to DMDSe resulted in induction of the expression of the ER oxidoreductase Ero1p with concomitant reduction of its regulatory disulfide bonds. These results suggest that MeSeH disturbs protein folding in the ER by generating a reductive stress in this compartment.

## 1. Introduction

During the last decades, extensive research has been focused on the identification of selenium (Se) compounds exhibiting cancer chemo-preventive properties [1]. Apart from prevention, redox active Se compounds have also been suggested to have anticancerous effects on many types of cancer [2,3]. Results from in vitro studies, animal experiments and clinical trials suggest that the biological activities of Se are dependent on the type and the nature of metabolites derived from the selenocompound under study [4].

Two key metabolites of Se, hydrogen selenide (H_2_Se), derived mainly from inorganic sources, and methylselenol (CH_3_SeH and MeSeH), derived from organic compounds, such as methylselenic acid (MSeA) and dimethylselenide (DMDSe), have been shown to be crucial for the anticancer properties of Se [5]. While the exact mechanisms of anticarcinogenic effects of Se derivatives are unknown, their cytotoxic effects are generally alleged to be caused by reactive oxygen species (ROS) produced by redox-cycling of redox active Se compounds with oxygen and thiols [6]. These redox cycles have been associated with DNA damage, thiol oxidation and oxidative stress, leading to cell cycle arrest and apoptosis [7,8,9]. For example, MeSeH, generated in vitro from selenomethionine (SeMet) and methioninase, was shown to redox cycle with GSH and oxygen, producing superoxides that were proposed to account for oxidative stress-induced cytotoxicity [10].

Additionally, MeSeH can act as a protein redox modulator targeting protein cysteine residues [11,12]. By using a thiol-proteomics approach, Park et al. [13] showed that MSeA, a commonly used MeSeH precursor, caused global thiol/disulfide redox modifications of numerous proteins distributed in various subcellular compartments. Redox modification of thiol/disulfide bonds may result in protein inactivation or protein misfolding and aggregation. Since the formation of disulfide bonds between cysteine residues occurs during the folding of many proteins that enter the secretory pathway, redox modifications of protein thiols may particularly affect the maturation of membrane-bound and secreted proteins in the endoplasmic reticulum (ER). Impaired homeostasis in the ER can lead to accumulation of misfolded proteins, which triggers a stress response called the unfolded protein response (UPR). This signaling pathway is under the control of the transmembrane protein inositol requiring kinase 1 (Ire1p), which coordinates the transcription of genes responsible for restoring ER homeostasis [14,15]. MSeA was, indeed, shown to induce ER stress and UPR activation in mammalian cancer cells [16,17,18,19,20].

In a previous paper, we studied the toxicity mechanisms of the MeSeH precursors MSeA, methylselenoglutathione (MeSeSG) and DMDSe in *Saccharomyces cerevisiae* [21]. We showed that MeSeH toxicity was mainly mediated by its metabolization into SeMet by O-acetylhomoserine (OAH)-sulfhydrylase followed by the conversion of SeMet into selenocysteine (SeCys) by the transsulfuration pathway resulting in SeCys misincorporation during protein synthesis, inducing toxic protein aggregation. This mechanism is not likely to account for MSeA anticancer effects because human cells lack OAH-sulhydrylase activity. However, at higher concentrations, MeSeH was toxic to mutant *met17* yeast cells which, like human cells, lack OAH-sulhydrylase activity and, therefore, may be considered as a model system to understand the effects of MeSeH in higher eukaryotes. We show that, in this strain, toxicity of the MeSeH precursor DMDSe is caused by an accumulation of misfolded proteins in the ER.

## 2. Results

### 2.1. Growth Inhibition by Methylselenol Precursors

**Figure 1:**
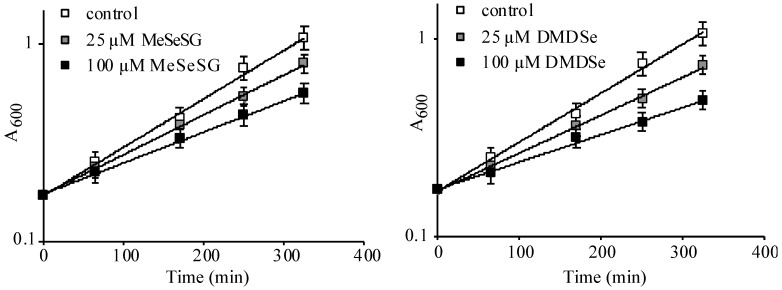
Cell growth inhibition by methylselenol (MeSeH) precursors. Exponentially growing BY4741 cells in SC medium supplemented with 100 µM methionine were diluted to 0.1 OD_600_ in the same medium and left to grow for 2 h. At time zero, 0 (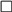), 25 (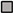) or 100 µM (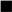) of methylselenoglutathione (MeSeSG) (**left panel**) or dimethyldiselenide (DMDSe) (**right panel**) were added and cell growth was monitored by measuring the OD_600_ at various times during 6 h. Data were plotted on a semi-log scale as a function of time. Doubling times of 119 ± 14 (control), 147 ± 16 (25 µM MeSeSG), 192 ± 19 (100 µM MeSeSG), 157 ± 16 (25 µM DMDSe) and 223 ± 24 (100 µM DMDSe) min were calculated by using regression analysis. The results are the mean and range of two experiments.

We, first, determined the concentration of MeSeSG or DMDSe necessary to inhibit the growth of BY4741, a *met17 S. cerevisiae* strain. Inside cells, both Se compounds can be reduced to MeSeH through nonenzymatic and enzymatic reactions [21]. Exposure to 25 µM DMDSe or MeSeSG reduced cell growth rate by 20–30%. At 100 µM, both compounds inhibited growth rate by 40–50% (Figure 1). These treatments did not affect viability as measured by a Trypan blue exclusion assay (>98% viable cells after 8 h in the presence of 100 µM DMDSe or MeSeSG). In addition, the vast majority of cells (>95%) retained colony-forming ability after 4 h exposures (result not shown).

### 2.2. Methylselenol Induces the Expression of Unfolded Protein Response (UPR) Targets

To gain insights into the mechanisms of MeSeH toxicity in *met17* cells, we used chromosomally integrated GFP-tagged constructs to study the expression of specific targets of transcription factors controlling stress responses, in cells exposed to DMDSe. We used *SSA4*, *TSA1* and *SOD1* and *KAR2* as fluorescent reporters for heat-shock, oxidative stress or ER stress, respectively. *SSA4* encodes a chaperone belonging to the HSP70 family of heat-shock proteins. Ssa4p expression is not detectable in normal growth conditions, but is upregulated by the transcriptional activator Hsf1p upon heat shock [22]. The thioredoxin peroxidase Tsa1p and the cytosolic superoxide dismutase Sod1p are targets of the transcription factors Yap1p and Skn7p [23], required for oxidative stress tolerance. Kar2p is an ER chaperone that binds to secretory and transmembrane precursor proteins to prevent their misfolding. Its expression is induced by the UPR. Kar2p is also involved in regulating the UPR through its interaction with the kinase/nuclease Ire1p [24]. To monitor Kar2p expression, we used a variant of GFP, called sfGFP, with improved folding capacity and a ER retrieval motif, that was previously shown to be correctly addressed to the ER [25].

**Figure 2:**
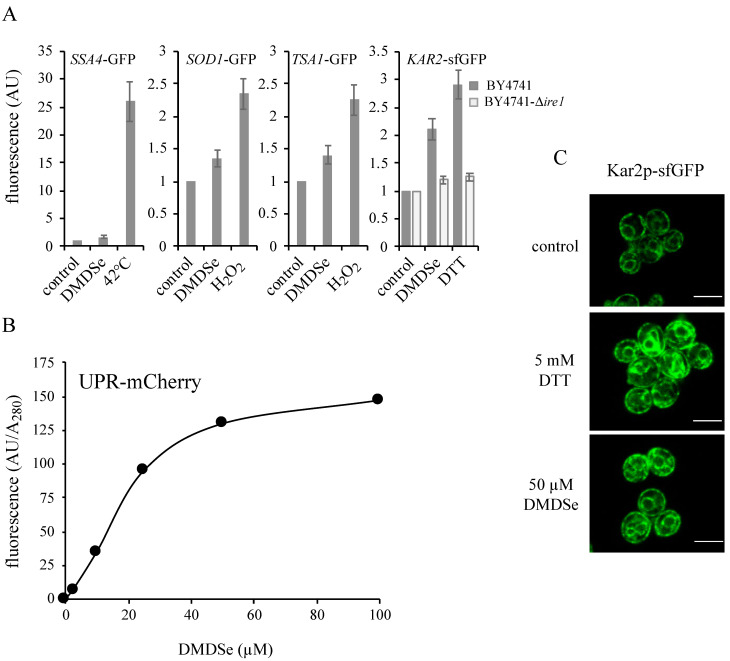
Cellular stress response to DMDSe exposure. (**A**) Fluorescence at 508 nm of whole cell extracts of BY4741 cells with a chromosomally integrated GFP-tagged construct, as indicated in the different panels, after incubation in SC + 100 µM methionine for 2 h at 30 °C (control) or 42 °C in the same medium, or at 30 °C in the additional presence of 100 µM DMDSe, 4 mM H_2_O_2_ or 5 mM DTT. The fluorescence of the Kar2p-sfGFP protein was determined in BY4741 (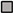) or in ∆*ire1* (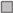) cell extracts. The fluorescence in whole cell extracts was recorded at 508 nm and normalized to the optical density of the extracts at 280 nm. The fluorescence intensity in the absence of toxic (control) was set as 1. The results are the mean ± S.D. of at least 3 experiments. (**B**) BY4741 cells expressing the fluorescent protein UPR-mCherry were grown in SC without uracil supplemented with 100 µM methionine. After incubation for 2 h with the indicated concentrations of DMDSe, the fluorescence in whole cell extracts was recorded at 610 nm. The autofluorescence of an extract from BY4741 cells in the absence of stress was subtracted from the results. (**C**) Kar2p-sfGFP localization was monitored by confocal fluorescence microscopy in living cells grown in SC + 100 µM methionine after 75 min of exposure to 50 µM DMDSe or 5 mM DTT. Bar equals 5 µm.

Figure 2A shows the fluorescence in crude extracts of cells exposed for 2 h to 100 µM DMDSe. A shift to 42 °C and exposures to 4 mM H_2_O_2_ or 5 mM DTT were used as positive controls for heat-shock, oxidative stress or ER stress, respectively. DMDSe failed to induce a heat-shock response in contrast to a temperature shift to 42 °C, indicating that protein homeostasis in the cytosol was not significantly disturbed by DMDSe. The expression of Tsa1p and Sod1p were slightly induced by exposure to DMDSe (1.3–1.5-fold) but less efficiently than by exposure to 4 mM H_2_O_2_ (2.1–2.4-fold). The Kar2p-sfGFP signal increased 2-fold after a 2 h treatment with DMDSe or nearly 3-fold with 5 mM DTT, suggesting that DMDSe caused ER stress. Since triggering UPR signaling in yeast strictly requires Ire1p, the UPR cannot be induced in ∆*ire1* cells. Our results show that the induction of Kar2p by DMDSe or DTT was dependent on the presence of Ire1p, since no change in Kar2p-sfGFP fluorescence intensity was observed in cell extracts of a ∆*ire1* strain (Figure 2A).

To confirm that DMDSe induced UPR activation, we used a previously described reporter gene encoding a fluorescent mCherry protein under the control of four tandem UPR elements, which is expressed only during ER stress [26]. As shown in Figure 2B, the fluorescence intensity of UPR-mCherry measured in crude extracts after 2 h of exposure increased with increasing DMDSe concentration and reached a maximum for cells incubated with 80–100 µM DMDSe.

The ER can be visualized using fluorescent intrinsic proteins such as Kar2p. It has been demonstrated previously that the ER expands during UPR induction in order to reduce the burden of unfolded proteins [27,28]. Imaging of live cells expressing Kar2p-sfGFP revealed a typical pattern of peripheral and nuclear ER fluorescence in unstressed cells. Upon DTT or DMDSe exposure, the intensity of fluorescence increased indicating the induction of UPR. In addition, ER membrane extension could be visualized (Figure 2C). Altogether, these results indicate that DMDSe/MeSeH induces an ER stress response.

### 2.3. Selenomethionine Impairs Protein Folding in the Cytosol But Not in the Endoplasmc Reticulum

**Figure 3:**
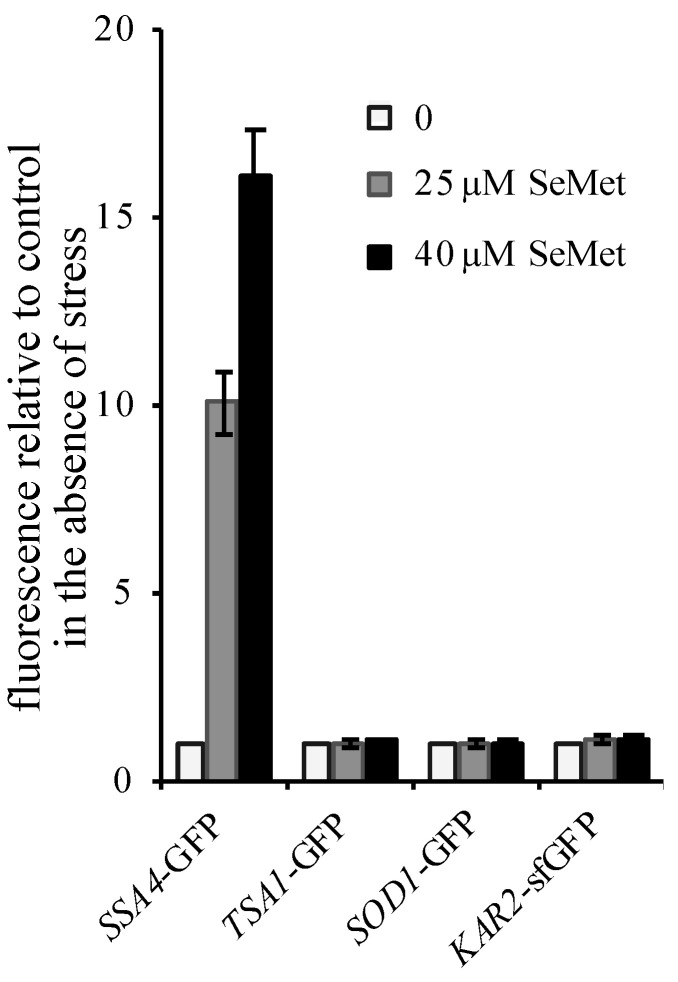
Cellular stress response to selenomethionine (SeMet) exposure. Exponentially growing BY4741 cells with chromosomally integrated GFP-tagged constructs were incubated in SC + 100 µM methionine for 2 h at 30 °C in the presence of 0 (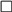), 25 (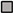) or 40 µM (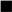) SeMet. The fluorescence in whole cell extracts was recorded at 508 nm and normalized to the optical density of the extracts at 280 nm. The fluorescence intensity in the absence of toxic was set as 1. The results are the mean ± S.D. of at least 3 experiments.

We have previously shown that SeMet exposure in yeast caused protein misfolding and aggregation in the cytosol and induced the expression of the protein chaperone Hsp104p under the control of the heat-shock factor Hsf1p [29]. To determine whether SeMet also impairs protein folding in the ER, we measured the fluorescence of the GFP-tagged constructs in cells exposed to SeMet (Figure 3). The results indicate that exposure to toxic concentrations of SeMet, resulting in a 20–50% reduction of the growth rate, increased the expression of *SSA4*, a target of Hsf1p, but was unable to trigger either an oxidative or ER stress response.

### 2.4. Null-Allele Strains of Genes Involved in the UPR Are Sensitive to Methylselenol but Not to Selenomethionine

**Figure 4:**
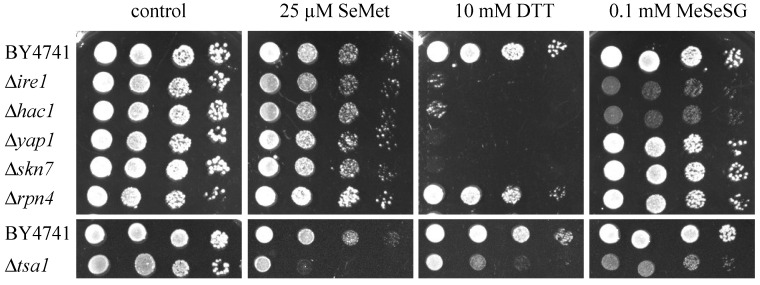
Sensitivity of yeast null-allele strains to SeMet, MeSeSG and DTT. Ten-fold serial dilutions of the indicated strains were spotted on solid SC + 100 µM methionine plates containing no toxic (control), SeMet (25 µM), DTT (10 mM) or MeSeSG (100 µM) and grown 48 h at 30 °C.

To determine the importance of different stress response pathways for survival in the presence of SeMet, MeSeH or DTT, we analyzed the tolerance of various deletion mutant strains to these compounds (Figure 4). As DMDSe is volatile, MeSeSG was used as MeSeH precursor in this experiment, which required growth on Petri dishes for several days.

∆*ire1* and ∆*hac1* cells who are unable to activate the UPR are hypersensitive to compounds causing ER stress such as DTT. We found that these strains were also sensitive to MeSeSG but not to SeMet. Our results confirm the DTT hypersensitive phenotype of ∆*yap1* and ∆*skn7* strains [30] whereas the deletion of these genes did not alter tolerance to MeSeSG or SeMet, indicating that the transcriptional activation of the oxidative stress response is not required for MeSeH resistance. ∆*tsa1* cells were sensitive to DTT and also to both Se compounds. Cells lacking *TSA1* were previously shown to be hypersensitive to SeMet [29] and to DTT [30]. *TSA1* encodes a protein acting both as a thioredoxin peroxidase catalyzing reduction of peroxides and as a molecular chaperone that binds unfolded proteins and prevents their aggregation [31]. Thus, sensitivity to DTT, SeMet and MeSeH may reflect the inability of the ∆*tsa1* mutant to cope with toxic protein aggregates in the ER or in the cytosol. We also tested the sensitivity of a strain deleted for *RPN4*, which codes for a transcription factor that stimulates expression of proteasome genes and is transcriptionally upregulated by the UPR. As shown in Figure 4, neither DTT nor Se stresses altered cell growth of this mutant, indicating that degradation of abnormal proteins by the proteasome is not a limiting factor for resistance to DTT- or MeSeH-induced ER stress.

### 2.5. Methylselenol Affects Oxidative Protein Folding in the ER

**Figure 5:**
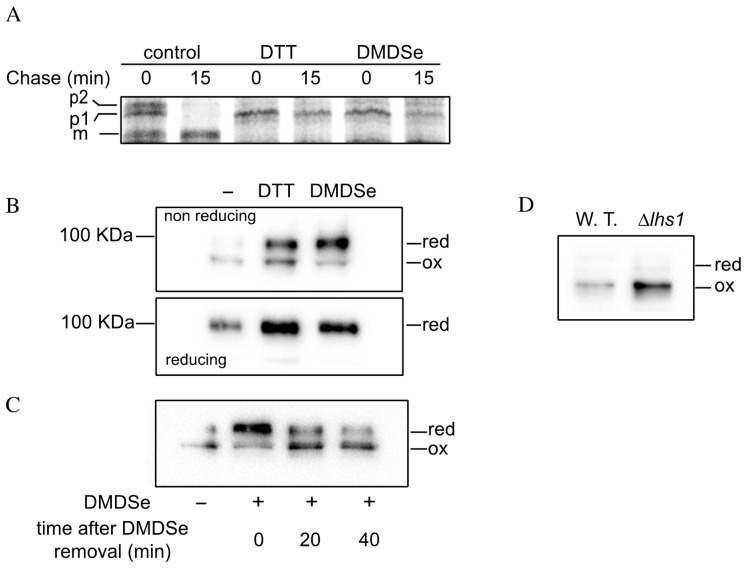
Disulfide formation in the ER is impaired by exposure to DMDSe. (**A**) Exponentially growing BY4741 incubated in SC + 100 µM methionine were exposed to nothing (control), 6 mM DTT or 100 µM DMDSe at 30 °C for 20 min before labeling for 10 min with the ^35^S-Protein mix in a medium without methionine. Cells were harvested after 0 or 15 min of chase with cold methionine and cysteine. Total proteins were extracted and Cpy1p was immunoprecipitated using anti-carboxypeptidase Y antibodies. Immunoprecipitated samples were analyzed by SDS-PAGE (8%), and detected using a Typhoon phosphorimager. The position of the ER (p1) or Golgi (p2) propeptides and the matured protein (m) is indicated. (**B**) BY4741 cells carrying pAF84 (2 μ *ERO1*-*myc URA3*) were grown in SD + 100 µM methionine. Cells were incubated in the absence or presence of 2 mM DTT or 100 µM DMDSe at 30 °C for 30 min. Free thiols were modified with iodoacetamide. Samples, containing equal amounts of proteins, were resolved by nonreducing SDS-PAGE (upper panel) or after the addition of 20 mM DTT in the sample buffer (lower panel). Ero1p-myc was detected by Western blotting with anti-myc antibodies. (**C**) BY4741 cells carrying pAF84 were incubated in the absence (−) or presence (+) of 50 µM DMDSe for 30 min, after which the incubation was continued in fresh medium without DMDSe. The oxidation state of Ero1p was assessed as in (**B**) in cell extract harvested either immediately, 20 or 40 min after DMDSe removal. (**D**) The oxidation state of Ero1p was assessed as in (**B**), in BY4741 cells (W.T.) or in a ∆*lhs1* isogenic strain carrying pAF84.

We tested whether DMDSe exposure impaired disulfide bond formation in the ER by following the maturation of carboxypeptidase Y (Cpy1p), a protein requiring formation of five disulfide bonds for proper folding and ER to vacuole transport. Cpy1p is produced as a 67 kDa precursor in the ER (p1 form). After folding and formation of disulfide bonds, it is glycosylated and processed to the Golgi (p2 form, 69 kDa) followed by transport to the vacuole where cleavage of the propeptide results in the mature 63 kDa form of the protein (m form). Cpy1p was shown to be retained in the ER of DTT-treated cells due to a defect in the formation of disulfide bonds [32]. We followed Cpy1p maturation by pulse–chase analysis in cells exposed to DTT or DMDSe. Cells were labeled with [^35^S]-Protein mix for 10 min and chased for 0 or 15 min. After immunoprecipitation with Cpy1p antibodies, the samples were subjected to SDS-PAGE and analyzed by autoradiography. As shown in Figure 5A, all three Cpy1p forms were detected immediately after the labeling period in unstressed cells. After the 15 min chase, only the mature vacuolar form was observed, indicating that the protein was correctly processed. In contrast, both DTT and DMDSe treatments resulted in Cpy1p retention in the ER, indicating that DMDSe/MeSeH impaired correct formation of disulfides.

Disulfide bonds are formed in the ER by dithiol/disulfide transfer reactions with the oxidized form of protein disulfide isomerase Pdi1p, which in turn is oxidized by the flavoprotein ER oxidase Ero1p [33]. Ero1p catalyzes the transfer of electrons from thiol substrates to molecular oxygen and is regulated through reduction and oxidation of regulatory disulfide bonds. When the ER balance shifts to overly oxidizing conditions, regulatory cysteine residues in Ero1p become oxidized to disulfides, which decreases Ero1p oxidase activity. In cells treated with DTT, Ero1p is converted to the reduced (active) form and then mostly returns to the oxidized (inactive) form after DTT removal [34].

We have previously shown that MeSeH formation from DMDSe is favored in the highly reducing conditions of the cytosol [21]. However, disulfide bond formation in the ER requires an oxidizing environment, which might favor spontaneous oxidation of MeSeH in DMDSe with concomitant production of ROS, resulting in hyperoxidizing conditions in the ER. Alternatively, like DTT, MeSeH is a reducing agent that might induce a reductive stress. To determine how the redox environment is disturbed by DMDSe/MeSeH, we monitored the redox state of the regulatory cysteine pairs of Ero1p. Since the regulatory bonds link distant residues of the polypeptide chain, the presence or absence of these bonds can be readily discerned by mobility differences on nonreducing SDS-PAGE. To determine the redox state of Ero1p in vivo, cells containing a plasmid expressing an Ero1p-myc protein were treated with DMDSe or DTT, lyzed with trichloroacetic acid (TCA) and incubated with iodoacetamide (IAA) to block free thiols. Ero1p was detected by Western blotting after nonreducing SDS-PAGE (Figure 5B). In the absence of stress, Ero1p was predominantly oxidized as reported previously [35]. Exposure to 2 mM DTT or 50 µM DMDSe led to the reduction of the regulatory disulfide bonds resulting in proteins with a lower mobility in SDS-PAGE. In cell lysates reduced prior to SDS-PAGE (Figure 5B, bottom panel), the increased intensity of the Ero1 polypeptide band in DTT or DMDSe stressed cells reflects the upregulation of *ERO1* by the UPR. When DMDSe was removed, Ero1p regulatory bonds reformed within 20–40 min (Figure 5C). To ensure that reduction/activation of Ero1p was a consequence of DMDSe exposure, and not caused by UPR induction itself, the redox state of Ero1p was analyzed in a ∆*lhs1* strain, in which UPR is constitutively activated. As shown in Figure 5D, in the absence of exogenous stress, ∆*lhs1* cell extracts displayed increased expression of the inactive oxidized form of Ero1p.

Altogether, these results suggest that cells exposed to DMDSe are subjected to a reductive ER stress preventing disulfide bond formation, resulting in accumulation of misfolded proteins in the ER and activation of the UPR.

## 3. Discussion

In this study, we explored the cellular stress response pathways activated by MeSeH precursors in a ∆*met17* yeast strain. We show that exposure to these compounds triggered the activation of the UPR. In addition, we observed that DMDSe exposure prevented disulfide bond formation in carboxypeptidase Y, causing ER retention of the proenzyme. These results suggest that MeSeH disturbs the redox homeostasis in the ER, which results in protein misfolding. The observation that cells unable to mount an ER stress response were hypersensitive to MeSeH indicates that ER stress plays a major role in MeSeH toxicity in ∆*met17* strains.

MeSeH precursors did not elicit a heat shock response in this yeast strain. This indicates that the protein folding machinery in the ER is more sensitive to thiol/disulfide modifications by MeSeH than its cytosolic counterpart. In contrast, SeMet exposure induced the expression of *HSP104* and *SSA4*, which are targets of Hsf1p ([29] and this study) but did not induce a ER stress response. Since SeMet toxicity is mediated by SeCys misincorporation in nascent polypeptides, this result suggests that cysteine replacement by SeCys imposes a lower burden on ER cysteine-containing proteins than on cytosolic proteins. Notably, the mechanism of toxicity described here is distinct from those already characterized for Se compounds in yeast: ROS-mediated DNA damage induced by exposure to H_2_Se and cytosolic protein aggregation caused by SeMet [29,36]. This toxicity mechanism is also different from that of MeSeH in wild-type yeast, where MeSeH is converted in toxic SeMet by OAH-sulfhydrylase.

Disulfide bond formation and isomerization is a crucial function of the ER. This process is achieved through a series of thiol-disulfide exchange reactions between substrate proteins and oxidoreductases Pdi1p and Ero1p [37]. The redox poise of the ER must be tightly controlled to allow for net formation of disulfide bonds, rearrangement of non-native bonds and reduction of terminally misfolded proteins destined for cytosolic degradation. Protein oxidative folding is exquisitely sensitive to redox perturbations of the ER lumen environment as both hyperoxidizing and reductive challenges were shown to activate ER stress signaling [38]. We show that Ero1p regulatory disulfide bonds were reduced upon exposure to DMDSe, suggesting that the redox state of the ER was shifted towards more reducing conditions. This result implies that ROS produced by redox cycling of MeSeH with oxygen do not significantly contribute to the toxicity of MeSeH in these yeast cells. This conclusion is strengthened by the observation that ∆*yap1* and ∆*skn7* mutants are not sensitive to DMDSe.

It may seem paradoxical that exposure of yeast cells to DMDSe, which is an oxidized compound, results in a reductive stress in the ER. In both the cytosol and the ER, GSH plays a major role in redox homeostasis. In *S. cerevisiae*, GSH biosynthesis and degradation take place in the cytosol, and glutathione reductase is present in the cytosol but not in the ER [39]. Thus, the GSH and GSSG pools in the ER are uncoupled from systems that govern GSH homeostasis in the cytosol. The GSH/GSSG ratio in the ER is much lower than in the cytosol [40] in order to promote formation of protein disulfide bonds. The major part of DMDSe is likely to be converted into MeSeH in the reducing environment of the cytosol. MeSeH is a small compound that can easily diffuse across membranes in its protonated form [41]. Moreover, MeSeH can efficiently reduce GSSG [21]. Thus, diffusion of MeSeH from the cytosol to the ER could increase the GSH/GSSG ratio in the ER and lead to a reductive stress in this compartment.

Methylselenol has been suggested to be a critical Se metabolite for anticancer activity [5]. Studies have shown that MeSeH can induce caspase-mediated apoptosis in cancer cells. Se precursors metabolized into MeSeH were shown to be potent tumor inhibitors. Moreover, in combination therapy, MeSeH-producing compounds were shown to enhance the efficacy of several cytotoxic drugs for cancer treatment [42]. Among several mechanisms proposed to account for MeSeH anticancer activity [42,43], a number of reports have shown that MeSeH-generating compounds, such as MSeA, induce growth arrest and apoptosis of cancer cells via UPR induction, activation of caspases and increased expression of proapoptotic genes such as C/EBP homologous protein (CHOP) [16,17,18]. Thus, activation of ER stress-induced apoptosis might be an important molecular mechanism underlying the anticancer activity of MeSeH. In mammalian cells, UPR is mediated by three ER transmembrane receptors: IRE1, activating transcription factor 6 (ATF6) and protein kinase R-like ER kinase (PERK). The corresponding signaling pathways act in concert to restore and maintain protein homeostasis in the ER. However, if the adaptive response fails upon prolonged ER stress, UPR signaling triggers apoptotic cell death [44,45].

To our knowledge, the present study is the first report showing that MeSeH induces ER stress by shifting the ER redox balance towards more reduced conditions. Provided that this mechanism can be extrapolated to human cells, this finding could help in the selection of drugs that act synergistically with MeSeH to inhibit growth of cancer cells.

## 4. Materials and Methods

### 4.1. Reagents

DMDSe (Sigma, Saint-Louis, MO, USA)) was prepared as a 20 mM stock solution in 100% ethanol and stored at −20 °C. MeSeSG was prepared according to [21]. L-SeMet and DTT were from Sigma. IAA and SDS were from BioRad (Hercules, CA, USA). Rabbit polyclonal anti-CPY antibodies were from Abcam (Cambridge, UK). Mouse monoclonal anti-myc antibodies (9E10) were from Sigma.

### 4.2. Strains and Media

The *S. cerevisiae* strains used in this study are derived from BY4741 (*MAT*a *his3*∆*1 leu2*∆*0 met15*∆*0 ura3*∆*0*). The parental strain and all the single mutants were obtained from Euroscarf (Scientific Research and Development GmbH, Oberursel, Germany). The BY4741 strains containing GFP-tagged constructs of *SSA4*, *TSA1* and *SOD1* were purchased from ThermoFisher Scientific (Waltham, MA, USA). Strains BY4741 Kar2p-sfGFP::HIS, BY4741 ire1Δ::KanMX4 Kar2p-sfGFP::HIS and BY4741 UPR-mCherry::URA were provided by P. Lajoie (University of Western Ontario, London, ON, Canada). The plasmid pAF84 (2 µ *URA3*-*ERO1*-*myc*) was obtained from C. Kaiser (MIT, Cambridge, MA, USA) and is described in [46]. Standard Synthetic Complete (SC) medium contained 0.67% (*w*/*v*) yeast nitrogen base (Difco, Franklin Lakes, NJ, USA), 2% (*w*/*v*) glucose and 80 mg/L of adenine, uracil and all amino acids (160 mg/mL leucine) except methionine and cysteine and was buffered at pH 6.0 by the addition of 50 mM MES-NaOH. Standard synthetic defined (SD) medium contained 0.67% (*w*/*v*) yeast nitrogen base, 2% (*w*/*v*) glucose and 50 mg/L of histidine, leucine and lysine and was buffered at pH 6.0 by the addition of 50 mM MES-NaOH.

### 4.3. Growth Assays

For growth inhibition assays, cells were grown overnight at 30 °C in SC medium supplemented with 100 µM methionine. Exponentially growing cells were diluted to 0.1 OD_600_ in the same medium and left to grow for 2 h, after which DMDSe or MeSeSG were added. Cell growth was monitored by measuring the OD_600_ at various times. For stress tolerance analysis of deletion mutants, cells grown overnight at 30 °C in SC medium supplemented with 100 µM methionine were diluted in water to obtain samples with optical densities of 0.5, 0.05, 0.005 or 0.0005 at 600 nm. Five microliters of each sample were spotted onto SC + 100 µM methionine agar plates containing 25 µM SeMet, 10 mM DTT or 100 µM MeSeSG. Plates were analyzed after incubation for 2 days at 30 °C.

### 4.4. Fluorescence Microscopy

BY4741 cells expressing Kar2-sfGFP were grown at 30 °C to an OD_600_ of 0.5 in SC medium supplemented with 100 µM methionine followed by exposure to 5 mM DTT or to 50 µM DMDSe for 75 min. Cells were mounted on glass slides covered with a thin layer of 1% (*w*/*v*) agarose. Confocal images were acquired at room temperature using a commercial confocal laser scanning microscope (TCS SP8, Leica, Wetzlar, Germany) equipped with an inverted frame (Leica), a high NA oil immersion objective (HC PL APO 63 × 1.40, Leica), and a white light laser (WLL, Leica). For the acquisitions we used the following settings: the excitation wavelength was set to 488 nm, the fluorescence was collected in the 500–550 nm band, the lateral pixel size was set to 92.35 nm, the line frequency to 400 Hz and the laser power to 16%.

### 4.5. Fluorescence Spectroscopy

Cells expressing GFP constructs were grown at 30 °C to an OD_600_ of 0.5 in SC medium + 100 µM methionine, followed by 2 h exposure to 100 µM DMDSe, 4 mM H_2_O_2_, 5 mM DTT, 25 or 40 µM SeMet or transfer to 42 °C. Whole cell extracts from 1 to 3 OD_600_ units were prepared in 100 mM potassium phosphate, pH 7.4, 10 mM MgCl_2_, 100 mM KCl, 10 mM 2-mercaptoethanol, by vortexing cells at 4 °C for 10 × 30 s in the presence of an equal volume of glass beads (500–750 µm). After centrifugation at 10,000× *g* for 10 min, the supernatant was recovered and the optical density at 280 nm was determined. GFP fluorescence was recorded at 508 nm in a Jasco FP-8300 spectrofluorometer using an excitation wavelength of 487 nm (bandwidth 2.5 nm). Cells expressing UPR-mCherry were grown in SC medium without uracil supplemented with 100 µM methionine and exposed to 0, 2.5, 10, 25, 50 or 100 µM DMDSe for 2 h. GFP fluorescence in whole cell extracts was recorded at 610 nm using an excitation wavelength of 580 nm (bandwidth 2.5 nm).

### 4.6. Analysis of Cpy1p Maturation

For carboxypeptidase Y maturation assays, BY4741 cells were grown at 30 °C to an OD_600_ of 0.5 in SC medium + 100 µM methionine. The culture was split in three and incubated with nothing, 6 mM DTT or 100 µM DMDSe at 30 °C for 20 min, harvested by centrifugation and resuspended at 5 OD_600_ units/mL in 0.8 mL SC medium lacking methionine. After a further incubation of 15 min, cells were pulse-labeled with 100 µCi (25 µCi/OD_600_) Express^35^S Protein Labelling Mix (Perkin Elmer-NEN, Waltham, MA, USA) for 10 min at 30 °C. Chase was performed by addition of 1 mM methionine and 1 mM cysteine. Cells (2 OD_600_ units) were collected in 20 mM NaN_3_, incubated at 4 °C for 10 min, resuspended in 200 µL of lysis buffer (50 mM Tris-HCl pH 7.5, 150 mM NaCl, 0.1 mM EDTA, 1% SDS, 1 mM phenylmethylsulfonyl fluoride (PMSF) and protease inhibitors (0.5 μg/mL aprotinin, 0.5 μg/mL antipain, 0.5 μg/mL chymostatin, 0.5 μg/mL leupeptin, 0.5 μg/mL pepstatin A, 100 μg /mL benzamidine, 1 μg/mL *o*-phenantroline and 6 μg/mL ovomucoid)) and lyzed by vortexing at 4 °C for 10 × 30 s in the presence of an equal volume of glass beads. After 3 min incubation at 92 °C, extracts were diluted to 1 mL in IP buffer (50 mM Tris-HCl pH 7.5, 150 mM NaCl, 0.1 mM EDTA, 0.1% triton X100, 0.5 mg/mL BSA, 1 mM PMSF and protease inhibitors). Proteins were immunoprecipitated with polyclonal anti-CPY antibodies in the presence of protein A sepharose magnetic beads (GE Healthcare) for 3 h at 4 °C on a rotative wheel. The beads were washed 4 times with 500 μL of IP buffer without BSA, resuspended in 15 μL of Laemmli buffer and incubated at 94 °C for 5 min. Samples were resolved by SDS-PAGE (8%), and analyzed with a Typhoon imager (GE Healthcare, Chicago, IL, USA).

### 4.7. In Vivo Oxidation State of Ero1p

To analyze the redox state of Ero1p, BY4741 cells containing the plasmid pAF84 were grown at 30 °C to an OD_600_ of 1 in SD medium + 100 µM methionine. The culture was split in three and incubated with nothing, 2 mM DTT or 100 µM DMDSe at 30 °C for 30 min. Cells (10 OD_600_ units) were lyzed by vortexing at 4 °C for 10 × 30 s in the presence of an equal volume of glass beads in 10% (*w*/*v*) TCA at 4 °C. Proteins were collected by centrifugation, washed once with chilled acetone and resuspended in 50 μL of non-reducing Laemmli buffer containing 25 mM IAA. Samples were incubated 30 min at 37 °C, followed by 2 min at 90 °C. For reduced control samples, 20 mM DTT was added to an aliquot of the sample before heating at 90 °C. Samples were resolved by nonreducing SDS-PAGE (7.5%) and Ero1p was detected by Western blotting with monoclonal anti-myc antibodies.

## Data Availability

Data is contained within the article.
